# Genome screen in familial intracranial aneurysm

**DOI:** 10.1186/1471-2350-10-3

**Published:** 2009-01-13

**Authors:** Tatiana Foroud, Laura Sauerbeck, Robert Brown, Craig Anderson, Daniel Woo, Dawn Kleindorfer, Matthew L Flaherty, Ranjan Deka, Richard Hornung, Irene Meissner, Joan E Bailey-Wilson, Carl Langefeld, Guy Rouleau, E Sander Connolly, Dongbing Lai, Daniel L Koller, John Huston, Joseph P Broderick, the FIA Study Investigators [Familial Intracranial Aneurysm Study Investigators]

**Affiliations:** 1 Indiana University School of Medicine, Indianapolis, IN, USA; 2 University of Cincinnati School of Medicine, Cincinnati, OH, USA; 3 Mayo Clinic, Rochester, MN, USA; 4 The George Institute for International Health, University of Sydney, Sydney, Australia; 5 Cincinnati Children's Hospital Medical Center, Cincinnati, OH, USA; 6 National Human Genome Research Institute, Baltimore, MD, USA; 7 Wake Forest University of Medicine, Winston-Salem, NC, USA; 8 Center of Excellence in Neuromics, University of Montreal, Montreal, Canada; 9 Columbia University, New York, NY, USA

## Abstract

**Background:**

Individuals with 1st degree relatives harboring an intracranial aneurysm (IA) are at an increased risk of IA, suggesting genetic variation is an important risk factor.

**Methods:**

Families with multiple members having ruptured or unruptured IA were recruited and all available medical records and imaging data were reviewed to classify possible IA subjects as definite, probable or possible IA or not a case. A 6 K SNP genome screen was performed in 333 families, representing the largest linkage study of IA reported to date. A 'narrow' (n = 705 definite IA cases) and 'broad' (n = 866 definite or probable IA) disease definition were used in multipoint model-free linkage analysis and parametric linkage analysis, maximizing disease parameters. Ordered subset analysis (OSA) was used to detect gene × smoking interaction.

**Results:**

Model-free linkage analyses detected modest evidence of possible linkage (all LOD < 1.5). Parametric analyses yielded an unadjusted LOD score of 2.6 on chromosome 4q (162 cM) and 3.1 on chromosome 12p (50 cM). Significant evidence for a gene × smoking interaction was detected using both disease models on chromosome 7p (60 cM; p ≤ 0.01). Our study provides modest evidence of possible linkage to several chromosomes.

**Conclusion:**

These data suggest it is unlikely that there is a single common variant with a strong effect in the majority of the IA families. Rather, it is likely that multiple genetic and environmental risk factors contribute to the susceptibility for intracranial aneurysms.

## Background

Subarachnoid hemorrhage due to the rupture of an intracranial aneurysm (IA) occurs in 16,000 to 17,000 persons in the U.S. annually and nearly half of affected persons are dead within the first 30 days. There are several important factors which modulate the risk of SAH. The incidence of SAH increases moderately with advancing age and it is the only stroke subtype in which women have a higher age-adjusted risk of SAH as compared to men [1]. Cigarette smoking has consistently been identified as the most important modifiable risk factor for SAH [2] with an average odds ratio of 3.1 [2,3]. In population-based and cohort studies, 70–75% of persons with SAH have a prior history of smoking and 50–60% are current smokers [2]. Hypertension accounts for an estimated 20% of all cases of SAH secondary to IA [2]. African-Americans have twice the age- and gender-adjusted risk of SAH as compared to whites [2].

In addition to the importance of environmental risk factors, studies have also consistently demonstrated a genetic component to the risk for SAH and IA. The risk for an SAH in first degree relatives of an SAH patient has been reported to range from 1.8 to as high as 6.6 times that of an age matched control [2,4-7]. Studies have also shown that first and second degree relatives of an SAH or IA patient are at increased risk for an unruptured IA (8.7% – 13.9%, compared to estimated 1% in the general population) [8-10].

Linkage analysis has been performed using both large families with evidence of a Mendelian form of IA [11-14] as well as large numbers of smaller families, typically with two affected, genotyped individuals [15-18]. While linkage to multiple regions of the genome has been reported, evidence to several chromosomes, 1p36, 5q31, 7q11, 14q22, 17cen, 19q13 and Xp22, has been found in more than one study. However, none of these studies considered environmental covariates when performing the linkage analysis. Recently, association of two SNPs on chromosome 9p21 has been reported in analyses consisting of cases and controls from Iceland, the Netherlands and Finland [19]. Analyses of each dataset individually as well as jointly found that the G allele at rs10757278 is associated with an approximately 1.29 fold increased risk of an IA.

We recently completed a mid-study genetic analysis of a sample of 192 multiplex IA pedigrees [20]. The greatest evidence of linkage was found on chromosomes 4q, 7q, 8q and 12q. We performed analyses including the average pack-years for the affected individuals in each family so as to detect significant gene × smoking interactions. Three of the four chromosomal regions (4, 7 and 12) all appear to have greater effect in those families with the heaviest smoking. Only on chromosome 8 did the inclusion of smoking as a covariate not significantly strengthen the linkage evidence, suggesting no interaction between the loci in this region and smoking. We have now genotyped the remainder of the sample and report linkage results in the full sample of 333 multiplex IA pedigrees. We have employed a series of complementary analyses allowing us to model both the disease locus as well as gene × smoking interactions. This approach increases the likelihood that we will detect loci contributing to the risk of IA.

## Methods

### Subjects

Probands with an intracranial aneurysm (IA) were identified by 26 clinical centers (with 41 recruitment sites) located throughout North America, New Zealand and Australia [21]. The Familial Intracranial Aneurysm (FIA) study was approved by the Institutional Review Boards/Ethics Committees at all clinical and analytical centers and recruitment sites. To be eligible, families were required to meet one of the following criteria: 1) at least 2 living affected siblings; 2) at least 2 affected siblings, one of whom is living and the other whose genotype could be reconstructed through the collection of closely related, living family members (i.e. spouse and children); 3) 3 or more affected family members (e.g. cousin, uncle, aunt), two of whom are alive and have living connecting relatives; or 4) 3 or more affected family members, with one living affected and at least one other affected relative whose genotype could be reconstructed through the collection of closely related, living family members. Exclusion criteria included: 1) a fusiform-shaped IA of a major intracranial trunk artery; 2) an IA which is part of an arteriovenous malformation; 3) a family history of polycystic kidney disease, Ehlers Danlos Syndrome, Marfan's Syndrome, fibromuscular dysplasia or Moya-moya disease; or 4) failure to obtain informed consent from the patient or family members.

Questionnaire data regarding demographics, environmental risk factors, and family history of IA was obtained from all family members. Blood was obtained for the isolation of DNA. The first degree relatives of affected family members who met study criteria for a higher risk of IA were offered a free, study MRA with time-of-flight sequences. The majority of MRAs were performed using a 1.5T (72.4%) MR imaging systems. The remainder were diagnosed with 3.0T (21.9%), 4.0T (0.9%) or were identified with CTA (4.8%). Unfortunately, we can not address the specificity and sensitivity of our MRA results because our patients did not proceed to DSA for validation of the non-invasive imaging tests. However, we note that MRA screening for IAs is standard medical practice and studies have reported MRA sensitivities and specificities between 90 and 97% for the detection of IAs [22,23]. Higher risk was defined as: 1) 30 years of age or older and 2) either had a 10 pack year history of current or former smoking; or had an average blood pressure reading of ≥ 140 mm Hg systolic or ≥ 90 mm Hg diastolic.

A Verification Committee reviewed all medical records and the phone screen of the proband and family members with a reported history of IA, SAH or intracerebral hemorrhage (ICH). Two neurologists who were members of the Verification Committee independently reviewed the subject's records and determined whether the subject met all the study inclusion and exclusion criteria. When the two members disagreed, a third neurologist reviewed the data. Each potential affected family member was ranked as definite, probable, possible, or not a case (Table 1).

**Table 1:** Disease phenotypes

**Classification**	**Definition**
Definite	Medical records document intracranial aneurysm (IA) on angiogram, operative report, autopsy, or a non-invasive imaging report (MRA, CTA) demonstrates an IA measuring 7 mm or greater.
Probable	Death certificate mentions probable intracranial aneurysm without supporting documentation or autopsy. Death certificate mentions subarachnoid hemorrhage (SAH) without mention of IA and a phone screen is consistent with ruptured IA (severe headache or altered level of consciousness) rapidly leading to death. An MRA documents an IA that is less than 7 mm but greater than 3 mm.
Possible	Non-invasive imaging report documents an aneurysm measuring between 2 and 3 mm. SAH was noted on death certificate, without any supporting documentation, autopsy or recording of headache or altered level of consciousness on phone screen. Death certificate lists 'aneurysm' without specifying cerebral location or accompanying SAH.
Not a Case	There is no supporting information for a possible IA.

### Genotyping

Genotyping was performed by the Center for Inherited Disease Research (CIDR) using the 6 K Illumina array. A total of 2,317 individuals from 394 families were genotyped by CIDR, with the samples sent at two intervals, with results from the first sample set previously reported [20]. All summary statistics reported herein are for the combined sample. The error rate, based on paired genotypes from 107 duplicate samples, was 0.0022%. The percentage of missing genotypic data was 0.24%

Extensive quality assessment of the genotypic data was performed prior to initiating any linkage analysis. The reported family structures were verified using the genotypic data [24] and, when necessary, pedigrees were altered. Significant changes in pedigree structure (n = 12 families) or changes in the initial disease status following phenotype verification (n = 49 families) resulted in the removal of 61 families based on their lack of informativeness for linkage analysis.

Once the family structures were finalized, the remaining marker data were reviewed in detail as a further step in data validation. Marker allele frequencies were estimated using PEDSTATS [25], which selected all Caucasian, non-Hispanic (the primary single ethnic group – 85% of the sample) unrelated individuals from which to estimate marker allele frequency. Markers or individuals were removed if: fewer than 90% of the individuals were genotyped for that marker (n = 28); fewer than 90% of the markers were genotyped for that individual (n = 2). Markers that violated Hardy Weinberg equilibrium at p < 0.001 (n = 4) or with very low informativeness (minor allele frequency < 0.05; n = 62) were removed from further analysis. Mendelian errors in each family were reviewed and genotypes were removed as needed to eliminate inconsistencies. Final genotypic quality control focused on removing any remaining genotypes that were likely to be erroneous as it is unlikely that recombination events would occur multiple times between adjacent markers [26]. A total of 16,799 genotypes (0.15% of the total) were removed in this final step.

### Statistical Analysis

Including markers in high linkage disequilibrium (LD) can inflate the evidence of linkage [27]; therefore, we computed pairwise LD (using both D' and r^2 ^statistics) and in all cases where D' exceeded 0.70, we retained the SNP with the highest minor allele frequency (i.e. the most informative marker). Analyses were also performed to assess the effect of employing r^2 ^as the measure of LD. In this instance, a threshold of r^2 ^> 0.40 was employed to identify SNPs with high LD. Results were similar using both thresholds and final SNP selection was based on results from the D' statistic [20].

The final analytic sample consisted of 333 families with 1,895 genotyped individuals. Of the 5,935 markers genotyped in common on the 6 K Illumina array by CIDR in the two independent samples, genotypic data from 4,839 markers were included in the final genome screen. Two models of disease were employed: 1) narrower disease definition, which classified as affected only those individuals who met the definite criteria (Table 1); and 2) broader disease definition, which classified as affected those individuals who met criteria for either definite or probable IA (Table 1).

We employed three complementary analytic methods to evaluate the evidence of linkage. First, we performed multipoint, model independent affected-relative pair linkage analysis as implemented in Merlin [28]. Using a multipoint approach allowed us to maximize the information content at each position in the genome. This is critical since each individual SNP has less power to detect linkage than does each microsatellite markers which is individually genetically more informative.

Second, we performed ordered subset analysis (OSA) to test for gene × smoking interactions [29,30]. A quantitative measure of cumulative smoking (pack-years) prior to the diagnosis of an aneurysm was computed for each affected individual in the genotyped families. Then, the average number of pack-years for each family was calculated using only the affected individuals. This calculation was performed separately for each of the two disease model definitions (narrow and broad). Then, families were ranked in descending order based on the average pack-years for the family. Multipoint, model independent affected-relative pair linkage analysis was then performed for the subset of families in the first rank (i.e. lowest average pack years) using the computer program Genehunter [30,31] and these linkage results were stored. The families in the next rank were then added to the first-rank families, and linkage analysis was performed again on this expanded set of families. This process was repeated until all families had been added to the analysis, at which point the "ordered subset" of families with the maximum LOD score (and the corresponding chromosomal position) were identified. Statistical significance of any observed increase in the maximum LOD score was determined using permutation methods by randomly ranking families. In the permutation procedure, the pack-years covariate values were randomly assigned to the families without replacement. The linkage analysis of ordered subsets was performed on this replicate as for the observed data, and the maximum LOD score on the chromosome was stored. 10,000 replicates were permuted in this fashion and a p-value for significance of the OSA result (LOD score as a function of smoking) was calculated by determining the proportion of these replicates that met or exceeded the observed OSA LOD score.

Given the extensive pedigree structures available for analysis, we proceeded to also perform parametric modeling. Similar to the previous analyses, no family members were classified as unaffected; all individuals not meeting disease criteria were coded as unknown. We avoided specifying disease parameters; rather we utilized an approach [32,33], which maximized the linkage evidence in each chromosomal region by estimating the disease parameters. This is often called a maximized LOD score (MOD score) and requires an upward adjustment in the typical significance thresholds employed in linkage analysis, since the disease parameters are being estimated and thus the test is fitting one additional parameter.

## Results

The narrower disease model included in the analysis 290 families consisting of 1,647 genotyped samples. There were 705 family members with definite IA. The broader disease model included 333 families with 1,895 genotyped samples, with 866 members meeting criteria for definite or probable IA (Table 2). Family structures varied widely and included both two generational and multigenerational pedigrees, with the largest pedigrees spanning five generations. Typically, the affected individuals were identified in either a single generation or in up to three generations. Overall, ~38% of the pedigrees were two generational, with most of these consisting of affected siblings. Another ~44% were three generational pedigrees with affected individuals in one, two or all three generations. Sixteen percent of pedigrees had four generations and the remaining 2% were five generations. Note that samples were not obtained in all generations; rather, these summary statistics describe the pedigree structure required to link genotyped individuals at-risk for disease.

**Table 2:** Sample demographics

	**Narrower Disease Model**	**Broader Disease Model**
Number of families	290	333
Number of genotyped individuals	1,647	1,895
% Caucasian, nonhispanic	84.5%	84.1%
		
*Individuals meeting criteria for IA*		
Total Number	705	866
% Female	76	75
Average age at diagnosis (years)	49.2	49.7
% current (prior) smoker^1^	47.9 (27.2)	48.4 (29.4)
Average pack years of smoking^1^	28.6	29.8
% reporting hypertension^1^	43.8	45.0
% reporting > 2 standard drinks per day^1^	9.8	9.6
		
*Individuals not meeting criteria for IA*		
Total Number	1025	1181
% Female	58	58
% current (prior) smoker^2^	28.6 (32.1)	27.3 (32.9)
Average pack years of smoking^2^	24.3	24.1
% reporting hypertension^2^	33.7	33.2
% reporting > 2 standard drinks per day^2^	8.9	9.1
		
# of all FIA family members undergoing MRA	318	380
% positive MRA (definite or probable)	2.8	7.6

^1 ^Data for the individuals with an IA is at the time of the identification of the aneurysm.

^2 ^Data for the individuals without an IA is at the time the questionnaire was completed.

MRA was offered to the subset of first degree relatives of affected individuals who also had a higher risk of IA due to age and smoking or hypertension. A study MRA was completed in 380 individuals from 182 different families. The average number of individuals who completed a study MRA in each family was 1.14; however, as might be expected the number of study MRAs in each family varied (range 0–8), and was influenced by the number of living members who met the high risk criteria.

**Figure 1:**
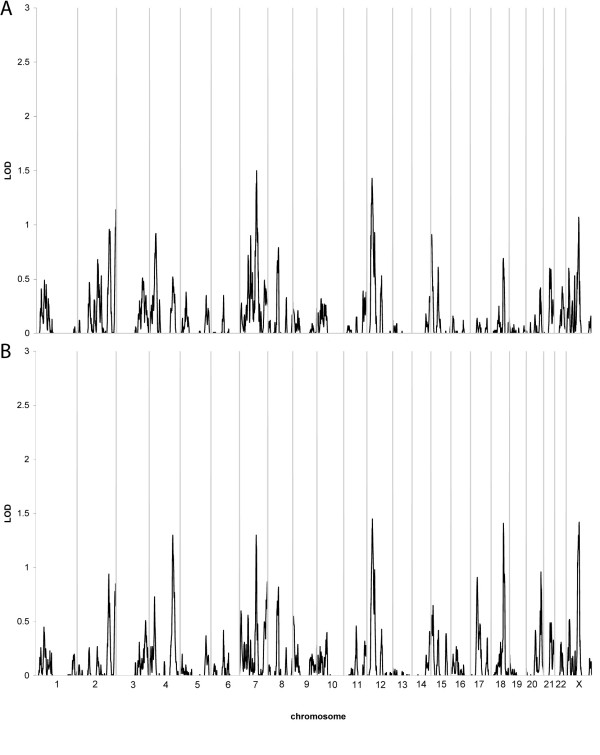
**Results of multipoint, model independent linkage analysis**. The X-axis depicts the various chromosomes across the genome with chromosome 1 at the far left and the X chromosome at the far right. The Y-axis indicates the LOD score at the various chromosomal positions across the genome. A. Narrower disease definition (n = 290 families); B. Broader disease definition (n = 333 families).

Genome-wide model independent linkage analysis employing both the narrower and broader disease model did not detect any chromosomal region with a LOD score greater than 1.5 (Table 3; Figure 1). We then performed the model independent linkage analysis using only the 283 families reporting themselves to be Caucasian as well as not Hispanic. When employing the narrower disease model, despite the smaller dataset, the possible evidence of linkage to chromosome 7 increased to a LOD = 1.75 near the marker rs441534. In all other regions, the evidence for linkage was slightly lower or unchanged. Similarly, when performing analyses with the broader definition, the LOD scores were slightly lower due to the smaller sample size, but otherwise relatively unaltered.

**Table 3:** Summary of linkage analyses

**Chromosome^1^/Position^2^**	**Model Independent Linkage Analysis** **LOD score**		**Ordered Subset Analysis** **Gene × smoking LOD score (p-value^3^)**		**Parametric Linkage Analysis** **LOD score**	
	
	**Narrower**^4^	**Broader**^5^	**Narrower**^4^	**Broader**^5^	**Narrower**^4^	**Broader**^5^
**4q32.3/160–162 cM**	0.5	1.3	NS^6^	NS	1.6	2.6
**7p14.1/60–63 cM**	0.7	0.6	4.1 (0.001)	3.2 (0.01)	0.4	0.7
**7q22.3/116–118 cM**	1.5	1.3	NS	NS	1.6	1.3
**12p12.3/35–50 cM**	1.4	1.4	NS	NS	2.4	3.1

^1 ^Cytogenetic position of the maximum LOD score

^2 ^Location of the maximum LOD score on the deCode map

^3 ^Permutation testing was performed to obtain p-values associated with the increase in LOD score observed when modeling gene × smoking interaction when compared with modeling for genetic effects only.

^4 ^Narrower disease model (n = 290 families)

^5 ^Broader disease model (n = 333 families)

^6 ^Not significant

Analyses were then performed to detect evidence for a gene × smoking interaction (Table 3). Evidence of a gene × smoking interaction was observed on chromosome 3p (Narrower model: 80 cM; p = 0.001; Broader model: 85 cM; p = 0.001) and two regions on chromosome 7, 7p14.1 (Narrower model: 63 cM; p = 0.001; Broader model: 60 cM; p = 0.01) and 7q36.2 (Narrower model: 188 cM; p = 0.008; Broader model: 167 cM; p = 0.01). However, for chromosome 3p and 7q, a small proportion of families provided the evidence for the interaction (typically 5–13% of the families). Therefore, since only a small proportion of the dataset contributed to these linkage results, these results are more likely to be spurious. We therefore focused our attention on the evidence of a gene × smoking interactions on chromosome 7p, which is supported by nearly a third of the sample, the 31% of families with the highest average rate of smoking in the affected subjects.

Finally, we performed parametric linkage analysis using the two disease definitions and maximized the disease parameters to obtain the greatest evidence for linkage. We identified two chromosomal regions of interest (Table 3). On chromosome 4q (162 cM), an unadjusted LOD score of 2.6 was obtained with the best fitting disease parameters including a rare disease allele (0.7% frequency) and reduced penetrance for both the heterozygotes (~25%) and homozygotes (~40%). On chromosome 12p (50 cM) an unadjusted LOD score of 3.1 was obtained. In this region, the maximal LOD score was obtained with a more common disease allele (4% frequency), and very low penetrance for heterozygotes (~5%) and modest penetrance for homozygotes (~50%).

## Discussion

We have performed a genome screen in the largest sample of multiplex IA families ever studied. With over 5,000 SNPs genotyped, we performed a series of complementary analyses to detect genetic factors which increase the susceptibility for IA. One of the major conclusions to be drawn from the multifaceted analyses performed in this study is that no chromosomal region provided strong evidence of linkage to IA. The sample size included in this study is relatively large and many of the families are quite extensive. Given the strong evidence regarding the heritability of IA, we interpret the modest results from the analyses of this large sample to suggest two possible hypotheses. The first hypothesis would postulate that multiple loci, rather than a single chromosomal region, contribute to the risk of IA and that several of these loci may have important interactions with other genes or environmental factors such as smoking. In this scenario, the presence of interactive effects rather than strong main effects, decreases the ability to detect linkage and also suggest that the combination of many different sets of loci may modify the risk for IA. The second hypothesis would be that there are some genes of strong effect, but that any one of these genes segregates in such a small proportion of the families that we do not have the power to detect their individual effects.

Despite the overall lack of a strong linkage signal with our multi-pronged approach, we were able to identify several chromosomal regions of potential interest. The region on chromosome 4q identified in this study was also detected in the previous analysis of the first portion of this dataset [20]. A nearby region (at 140 cM) was also reported linked in a sample of 119 families with at least two members with an abdominal aortic aneurysm [34]. A region on chromosome 12p was also identified. We previously detected evidence of linkage to chromosome 12 in a subset of the families analyzed in the current dataset [20]. Importantly, in the previous study, possible evidence of linkage was found in a region on chromosome 12q21.33 (at 102 cM). Analyses to detect gene × smoking interaction found the greatest effect of this locus in families with the heaviest rates of smoking. The data we report in this enlarged sample would appear to have detected a completely distinct locus on chromosome 12p, more than 50 cM proximal to the previously reported locus. We also found some possible support for linkage to chromosome 7q22. A region on 7p14 provided the strongest evidence for a gene × smoking interaction. Approximately one third of the families, all heavy smokers, contributed to this interaction. Thus, as we have enlarged the sample from 192 families to 333 families, we have continued to detect linkage to chromosome 4q; however, there are several other regions identified in the earlier study that were not detected in the new analyses. There are several possible reasons for this. The first is that the initial finding may have been a false positive result and with the larger sample size, we have greater power to detect true linkage. Another possibility is that with the addition of over 100 new families, we have not included the same genetic risk factors that were segregating in the initial group of families. It is impossible to test this hypothesis; however, evidence against this possibility is that families continue to be recruited at the same sites and there is no obvious difference in the clinical findings or family history of the previously analyzed families and the newly analyzed families. With these caveats, the primary advantage of the new analysis is the much larger sample of families included in the analysis, providing greater power to detect linkage.

The analyses performed in this large dataset did not detect evidence of linkage to the chromosomal regions identified in more than one previous study: 1p36, 5q31, 7q11, 14q22, 17cen, 19q13 and Xp22. Our study, as well as previous linkage studies, did not detect evidence of linkage to chromosome 9p21 [19] which was recently reported associated to both IA and abdominal aortic aneurysm in multi-ethnic samples (odds ratio of 1.29). Linkage studies, even one with over 300 families as this one has, does not have the power to detect a locus with such a small effect size, even if the SNP allele is relatively common, as is the case with the high risk G allele of rs10757278.

We have explored the effect of gene × smoking interactions in the FIA Study sample. As shown in Table 2, nearly three quarters of the individuals meeting criteria for IA are either current or past smokers. Therefore, this study has far greater power to detect loci which interact with smoking and quite limited power to detect loci that act only in those individuals who do not smoke. For this reason, we focused our interaction analyses toward the identification of loci having their greatest effect in families with greater smoking (i.e. higher pack years).

This study has several advantages. First, a large number of multiplex IA families were ascertained through an international consortium. This series of families has the power to detect loci with strong to moderate genetic effects. Second, uniform collection and review of clinical and environmental exposure data was performed improving the power to detect genetic effects. Third, individuals within the families at increased risk for an IA were offered MRA, allowing us to further improve the power to detect linkage by increasing the number of affected individuals in the pedigree. Fourth, a 6 K SNP screen was performed reducing the likelihood that any region of the genome would be uninformative and again maximizing the power to detect linkage anywhere in the genome.

This study also had potential limitations. In order to accrue the large number of families analyzed in this dataset, families were recruited from a wide geographic region across two continents. This could result in a heterogeneous sample with many different susceptibility genes segregating within the sample. Some other studies have focused on only one or a small number of families, typically from the same geographic or ethnic origin. This more limited approach would likely limit the number of different susceptibility genes segregating in the sample and could increase the power to detect the genes contributing to IA in that small number of families. To reduce the potential heterogeneity in the dataset, we performed our model-independent linkage analysis in a more limited dataset consisting of only those families reporting themselves to be Caucasian and not Hispanic. Results were relatively unaltered suggesting that the linkage findings were primarily supported by the Caucasian non-Hispanic families which represent the vast majority of our study families. Another limitation of this study may be a bias introduced by offering a study MRA to only higher risk family members. For cost savings, a study MRA was only offered to study participants who had a critical risk factor (smoking, hypertension) that increased the likelihood that an unruptured aneurysm might be identified. Therefore, it is possible that aneurysms detected by study MRA may be more likely to be the result of a critical environmental exposure (smoking) rather than genetic factors. However, given the small number of positive MRA which met criteria for Probable or Definite criteria (< 9%), it is unlikely that the statistical results would be significantly biased by the inclusion of a small number of potentially environmentally influenced cases.

The use of dense SNP marker maps, such as those currently available for genome wide association studies, may be an important tool needed to identify the genetic risk factors influencing IA susceptibility. We are currently performing a case control study I the FIA sample with a dense set of SNP markers to improve our power to detect common risk alleles with moderate effect.

## Conclusion

These data suggest it is unlikely that there is a single common variant with a strong effect in the majority of the IA families. Rather, it is likely that multiple genetic and environmental risk factors contribute to the susceptibility for intracranial aneurysms.

## Competing interests

The authors declare that they have no competing interests.

## Authors' contributions

TF contributed to the conception and design of the study and the statistical analysis and interpretation of data. LS contributed to the conception and design of the study and data acquisition. RB contributed to the conception and design of the study and the interpretation of the imaging data. CA contributed to the conception and design of the study and data acquisition. DW contributed to the conception and design of the study and phenotype verification. DK contributed to the conception and design of the study and phenotype verification. MLF contributed to the conception and design of the study and phenotype verification. RD contributed to the conception and design of the study and the interpretation of statistical analyses. RH contributed to the conception and design of the study. IM contributed to the conception and design of the study and interpretation of imaging data. JEB-W contributed to the conception and design of the study and interpretation of statistical analyses. CL contributed to the conception and design of the study and interpretation of statistical analyses. GR contributed to the conception and design of the study and data acquisition. ESC contributed to the conception and design of the study and data acquisition. DL contributed to the statistical analysis of the data. DLK contributed to the conception and design of the study and statistical analyses. JH contributed to the conception and design of the study and interpretation of imaging data. JPB contributed to the conception and design of the study, phenotype verification and data acquisition. All authors helped to draft the manuscript and read and approved the final manuscript.

## Appendix I

Clinical Centers – University of Alabama at Birmingham: W. Fisher, (PI), H. Forson, coordinator; Clinical Trials Research Unit, University of Auckland and Auckland City Hospital, New Zealand: C. Anderson, (PI), E. Mee, (PI), C. Howe, coordinator, S. Vos, coordinator; Royal Perth Hospital, Sir Charles Gairdner Hospital, Royal Adelaide Hospital, Royal Melbourne Hospital, Alfred Hospital, Westmead Hospital, Royal North Shore Hospital, Royal Prince Alfred Hospital, Australia: C. Anderson, (PI), G. Hankey, (PI), N. Knuckey, (PI), J. Laidlaw, (PI), P. Reilly, (PI), N. Dorsch, (PI), M. Morgan, (PI), M. Besser, (PI), J. Rosenfeld, (PI), K. Athanasiadis, coordinator, A Claxton, coordinator, V. Dunne, coordinator, J. Griffith, coordinator, J Davidson, coordinator, S. Pope, coordinator, Amanda Froelich, coordinator; Brigham & Women's Hospital: A. Day, (PI), R. Brach, coordinator; University of Cincinnati: D. Woo, co-(PI), M. Zuccarello, co-(PI), A. Ringer, co-(PI), H. Yeh, co-(PI), K. Franklin, coordinator; Cleveland Clinic Foundation: P. Ramussen, (PI), D. Andrews-Hinders, coordinator, T. Wheeler, coordinator; Columbia University: E. S. Connolly, (PI), R. Sacco, co-(PI), D. LaMonica, coordinator; University of Florida: S. B. Lewis, (PI), A. Royster, coordinator; Indianapolis Neurosurgical Group: T. Payner, (PI), N. Miracle, coordinator; Johns Hopkins: K. Murphy (PI), B. Kohler, coordinator; Massachusettes General Hospital: C. Ogilvy, (PI), D. Buckley, coordinator, J. Manansala, coordinator; London Health Science Center Research Inc.: G. Ferguson, (PI), C. Mayer, coordinator, J. Peacock, coordinator; Notre Dame Hospital: G. Rouleau, (PI), A. Desjarlais, coordinator; University of Maryland: E. F. Aldrich, (PI), C. Aldrich, coordinator, C. Byard, coordinator; Mayo Clinic: R. D. Brown (PI), L. Jaeger, coordinator; University of Michigan: L. Morgenstern, (PI), M. Concannon, Coordinator; New Jersey Medical School: A. I. Qureshi, (PI), P. Harris-Lane, coordinator; Northwestern University: H. Batjer, (PI), G. Joven, S. Thompson, coordinator; University of Ottawa: M. T. Richard, (PI), A. Hopper, (PI); University of Pittsburgh: A. B, Kassam, (PI), K. Lee, coordinator; University of California, SF, C. Johnston, (PI), K. Katsura, coordinator; University of Southern California: S. Giannotta, (PI), D. Fishback, coordinator; Stanford University Medical Center: G. Steinberg, (PI), D. Luu, coordinator, M. Coburn, coordinator; University of Texas at Houston: M. Malkoff, (PI), A. Wojner, coordinator; University of Virginia: N. Kassel, (PI), B. Worrall, co-(PI), G. Radakovic, coordinator; University of Washington: D. Tirschwell, (PI), P. Tanzi, coordinator; Washington University: C. Derdeyn, (PI), M. Catanzaro, coordinator; University of Manitoba (Winnipeg), A. Kaufmann, (PI), D. Gladish, coordinator.

## Pre-publication history

The pre-publication history for this paper can be accessed here:


